# Assessment of whole gut motility in adolescents using the wireless motility capsule test

**DOI:** 10.1007/s00431-021-04295-6

**Published:** 2021-11-17

**Authors:** Tanja Fritz, Christoph Hünseler, Ilse Broekaert

**Affiliations:** 1 Department of Paediatrics, Faculty of Medicine, University Children’s Hospital, University of Cologne, Cologne, Germany; 2 Cologne, Germany

**Keywords:** Functional gastrointestinal disorders, Gastrointestinal motility, Paediatric patients, Wireless motility capsule

## Abstract

Functional gastrointestinal (GI) disorders are often associated with intestinal dysmotility representing a diagnostic challenge. A relatively new method is the wireless motility capsule (WMC) test, which continuously measures pH, pressure, temperature and regional transit times as it passes through the GI tract. In adults, the WMC test was approved for use in the diagnosis of gastroparesis and constipation by assessing GI transit and contractility. We performed the WMC test in nine adolescent patients aged 12–17 years with functional GI symptoms from July 2017 until February 2019. Abnormal transit times were detected in four patients. Three patients showed abnormal transit times of the upper GI tract: in two cases, contractility analysis revealed prolonged gastric retention, and in one patient, abnormal colonic transit was detected.

*Conclusion*: The WMC test is a minimally invasive procedure with potential to expand future diagnostic opportunities for paediatric patients with functional GI disorders and suspected motility disturbances.

**Table:** 

**What is Known:** *• The assessment of GI transit and contractility of the whole gut is possible with the WMC test which is approved for use in the diagnosis of gastroparesis and constipation in adults. *	
**What is New:** *• The WMC test is a non-invasive diagnostic tool with the potential to expand diagnostic opportunities in paediatric patients by assessing regional and whole gut motility.* *• In paediatric patients with functional GI disorders, the WMC test could help to make an adequate diagnosis and initiate appropriate therapy.*

**Supplementary information:**

The online version contains supplementary material available at 10.1007/s00431-021-04295-6.

## Introduction

Functional gastrointestinal (GI) disorders are often associated with visceral hypersensitivity and dysmotility. Several methods for assessing GI motility (e.g. antroduodenal or colonic manometry) or GI transit (e.g. scintigraphy) are available but are invasive or lead to radiation exposure. A relatively new, non-invasive method is the wireless motility capsule (WMC) test [1] analogously to the wireless capsule endoscopy which is routinely used in, e.g. inflammatory bowel disease or gastrointestinal bleeding. After swallowing, the motility capsule continuously measures pH, pressure and temperature and enables to determine regional transit times as it passes through the entire GI tract. In adults, the WMC test has been approved for the assessment of regional and entire GI transit times, the evaluation of gastric emptying in gastroparesis and colonic transit in constipation [2, 3]. To date, data on WMC in paediatric patients are rare. Green et al. showed a sensitivity of 100% using the WMC test compared to scintigraphic gastric emptying studies to detect gastroparesis in 22 paediatric patients [4]. Rodriguez et al. found no association between the WMC study and symptoms but a fair agreement with gastric scintigraphy and a strong agreement with colonic radiopaque marker studies [5].

As in scintigraphic studies, regional transit times can be measured by the motility capsule. This is due to the fact that its pH sensor detects pH changes assigned to a specific GI region. The WMC test is applied according to a standardised protocol, whereas scintigraphic emptying study protocols can vary [6]. Antroduodenal or colonic manometry detects motility disorders of the upper or lower GI tract giving a very detailed profile of pressure pattern, including contraction frequency, amplitude and propagation [7]. To date, the availability of this method is restricted to specialised paediatric centres. With the WMC test, it is possible to analyse the motility pattern of specific regions in one measurement, like for instance the gastroduodenal region to define gastroparesis [8, 9] or the colonic region to define constipation [10, 11]. In paediatric patients, the WMC test has been shown to be even more sensitive than antroduodenal manometry (ADM) in detecting motor abnormalities [4].

With regard to diagnostic utility, the WMC test has been able to provide new diagnostic results in approximately 50% of adult patients with suspected dysmotility leading to a change in therapy [12, 13]. By examining the entire GI tract with the WMC test, abnormal regional transit times were not only detected in the presumably affected region, but also in other regions [13]. Thus, more targeted therapies (e.g. prokinetics or laxatives) could be used, especially in cases where symptoms did not predict the underlying pathology [14].

The aim of this data analysis was as follows:

i. to confirm the feasibility, safety and utility of the WMC test and to share our experience as there is little data on the WMC test in the paediatric population to date and
ii. to assess if the WMC could contribute to an individualised therapy concept especially in the case of functional GI disease where diagnostic possibilities are limited.

## Methods

A retrospective data analysis of WMC test data was performed. In total, data of nine adolescent patients with functional GI disorders (i.e. chronic abdominal pain, nausea, functional dyspepsia, constipation) and possibly affected gastrointestinal motility from the Paediatric Gastroenterology outpatient clinic at the University Hospital of Cologne, Germany, who underwent the WMC test between July 2017 and February 2019 were included for further analysis (Table 1). The diagnosis of each functional GI disorder was made according to Rome IV criteria and detailed clinical evaluation and examination, laboratory investigations and ultrasound were performed in all patients prior to the WMC test. In selected patients, additional motility testing as the radiopaque marker study was performed prior to the WMC test.

**Table 1:** Summary of WMC test results including regional gut transit times and contractility parameters calculated for the gastric and the small bowel region

No	Sex	Age	Dx	Transit times					Contractility parameter					
									Gastric window			Small bowel window		
				GET	SBTT	CTT	SLBTT	WGTT	Ct	MI	AUC	Ct	MI	AUC
				< 5 h	< 6 h	< 59 h	< 64 h	< 73 h	> 0.48 min^−1^	> 9.82	> 1358 mmHg s^−1^	> 0.6 min^−1^	> 10.57	> 1456 mmHg s^−1^
*Patients with normal transit*														
1	f	16y	IBS	3.44	4.11	14.52	19.04	22.48	0.46	44.94	2636	0.55	17.98	1072
2	m	12y	AM	4.08	3.4	15.47	19.27	23.36	0.89	37.25	2185	0.9	28.61	1678
6	f	16y	IBS	2.35	4.27	23.37	28.05	30.4	0.14	7.25	246	0.46	14.03	800
4	m	14y	FC	2.14	6.0	12.05	18.07	20.22	4.11	97.28	5773	3.09	82.27	3867
7	m	12y	N/V	2.27	5.1	41.19	46.3	48.57	1.98	42.88	2015	1.83	41.89	2220
*Patients with abnormal transit (GET; SBTT; CTT)*														
3	f	15y	FD	5.04	6.29	23.22	29.51	34.56	0.13	15.43	802	1.16	26.44	1578
9	f	17y	N/V	13.44	12.21	18.33	30.55	44.4	0.58	61.43	3522	0.2	5.28	313
5	m	12y	IBS, FC	0.43	7.02	50.05	57.07	57.5	1.71*	32.5*	673*	5.33	124.5	5727
8	m	15y	FD, FC	3.5	8.56	102.37	111.34	115.24	0.7	111.8	3987	1.06	41.35	2026
*30 min before gastric emptying														
			Median	3.4	6.0	23.2	29.5	34.6	0.6	43.9	2411	1.1	28.6	1678
			IQR 25	2.3	4.3	15.5	19.3	23.4	0.4	31.8	1712	0.6	18.0	1072
			IQR 75	4.1	7.0	41.2	46.3	48.6	1.2	70.4	3638	1.8	41.9	2220

*Dx*, diagnosis; *GET*, gastric emptying time; *SBTT*, small bowel transit time; *CTT*, colonic transit time; *SLBTT*, small and large bowel transit time; *WGTT*, whole gut transit time; *Ct*, number of contractions per minute; *MI*, motility index; *AUC*, area under the pressure curve; *IBS*, irritable bowel syndrome; *AM*, abdominal migraine; *GP*, gastroparesis; *FC*, functional constipation; *N/V*, nausea and vomiting; *FD*, functional dyspepsia; *IQR*, interquartile range

After exclusion of contraindications for the WMC test such as swallowing difficulties, inflammatory bowel disease or history of bowel surgery with possible GI obstruction, written informed consent was obtained from all patients and caregivers. Medications possibly affecting GI motility were discontinued at least 3 days before the WMC test and no bowel management was carried out before capsule ingestion.

The WMC test was performed according to the manufacturer’s recommendations (SmartPill®; SmartPill Corporation, Buffalo, NY, USA). After an overnight fast, the patient ingested a standardised meal (260-kcal nutrient bar, SmartBar) just before swallowing the capsule with a glass of water. After starting the measurement, a 6-h fasting period followed. The patient wore the recording data receiver for 5 days and documented any symptoms or activities. After excretion of the capsule (accompanied by loss of connection to the receiver) or after approximately 120 h when the battery was flat, the recorded data were uploaded from the receiver via the corresponding manufacturer’s test software (Supplemental Fig. 1a).

**Fig. 1:**
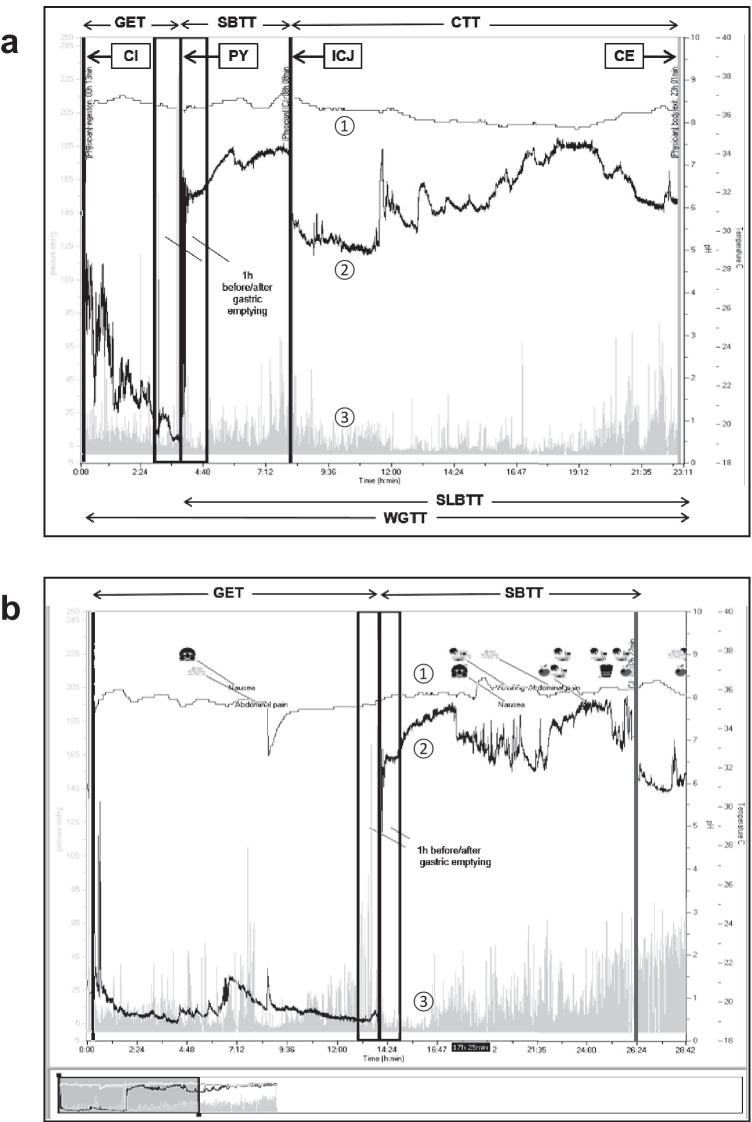
Gut transit profiles determined by WMC test. **a** Healthy patient; **b** patient with gastroparesis showing prolonged GET and SBTT. ①, temperature curve; ②, pH curve; ③, pressure columns. CI, capsule ingestion; PY, pylorus transit; ICJ, ileocecal junction; CE, capsule excretion

The WMC test continuously measured temperature, pH and pressure to calculate gastric emptying time (GET), small bowel transit time (SBTT), small and large bowel transit time (SLBTT), colonic transit time (CTT) and whole gut transit time (WGTT). GET was defined as time from ingestion, indicated by an increase of temperature and a pH decrease (norm < 4) when the capsule reaches the stomach until pyloric passage into the duodenum. This was determined by an abrupt pH increase (norm 2–4 pH units) from the lowest postprandial value. SBTT was defined as the time interval between capsule entry into the small bowel and the passing of the ileocecal valve indicated by a pH decrease of > 1 unit. CTT was defined as the time between the end of SBTT and excretion of the capsule, indicated by an abrupt temperature and pressure drop. SLBTT and WGTT can be calculated, respectively (Fig. 1) [10, 15, 16]. The reference ranges of transit times and contractility parameters have not been established in the paediatric population so far, therefore, adult reference ranges were used. A GET > 5 h and an SBTT > 6 h were considered prolonged. Colonic transit time was defined as normal < 59 h and the capsule should be excreted within 72 h (WGTT) [10]. Moreover, for the gastric and small bowel region, additional contractility parameters such as motility index, contraction frequency (contractions per minute; Ct/min), area under the curve (AUC) and motility index (MI) were calculated using the Software GIMS Data Viewer, version 3.0.0.

In the context of this publication, the WMC test results were pseudonymised for further retrospective data analysis. To summarise measures of the WMC test, descriptive statistics was used. WMC test data were described using median and interquartile ranges (IQR) (Table 1).

## Results

A complete dataset of the WMC test results was obtained from nine patients (median age 15 years; IQR 25–75: 12–16 years) from July 2017 until February 2019. All patients had functional GI symptoms suggestive for GI dysmotility (Tables 1 and 2). The WMC test was implemented without complications (problems of swallowing or capsule retention, etc.). Capsule measurement data including a symptom diary was obtained from all nine patients.

**Table 2:** Impact of WMC test results on diagnostic information, medication and additional diagnostic tests in patients with delayed transit times

**No**	**Symptoms**	**Dx**	**Medication**	**Diagnostic tests**	**WMC test**	**New diagnostic information**	**New medication**	**Additional diagnostic tests**	**Outcome**
3	Epigastric pain, nausea, vomiting	FD	PPI, peppermint oil	Upper GI endoscopy, WMC test	GET 5.04 h (< 5 h), SBTT 6.29 h (< 6 h)	Suspected gastroparesis, small bowel transit delay	UDCA, PPI, prokinetics	None	Improvement of symptoms
5	Abdominal pain, constipation	IBS FC	Macrogol	WMC test	SBTT 7.02 h (< 6 h) CTT 50 h (< 59)	Small bowel transit delay	Psyllium husks, macrogol psychotherapy	MRI, rectal biopsies	Improvement of symptoms
8	Epigastric pain, postprandial fullness, constipation	FD FC	Probiotics, PPI, macrogol, prokinetics	MRI, upper GI endoscopy, WMC test	SBTT 8,5 h (< 6 h) CTT 102 h (< 59)	Small bowel transit delay, slow transit colon	UDCA, PPI, macrogol, psyllium husks, probiotics, prokinetics	Contrast meal and follow-through	Improvement of dyspepsia, persistent constipation
9	Recurrent nausea and vomiting, regurgitation, abdominal pain	N/V	NaSSA, PPI,	Upper GI endoscopy, contrast meal and follow-through, WMC test	GET 13.44 (< 4 h), SBTT 12.21 (< 6 h)	Gastroparesis, small bowel transit delay	Probiotics, prokinetics	None	Significant relief of symptoms

*Dx*, diagnosis; *GET*, gastric emptying time; *SBTT*, small bowel transit time; *CTT*, colonic transit time; *IBS*, irritable bowel syndrome; *FC*, functional constipation; *N/V*, nausea and vomiting; *FD*, functional dyspepsia; *PPI*, proton pump inhibitor; *UDCA*, ursodeoxycholic acid; *PEG*, polyethylene glycol; *ADM*, antroduodenal manometry; *MRI*, magnetic resonance imaging; *NaSSA*, noradrenergic and specific serotonergic antidepressant

### Regional gut transit times and pH

Recording of regional transit times revealed a median GET of 3.4 h (IQR 2.3–4.1) (norm < 5 h), a median SBTT of 6.0 h (IQR 4.3–7.0) (norm < 6 h) and a median CTT of 23.2 h (IQR 15.5–41.2) (norm < 59 h).

In total, four of nine patients showed abnormal transit times in one or more gut regions. Two of these patients showed both a prolonged GET and a prolonged SBTT (Table 1). In one patient with severe chronic recurrent vomiting, GET was 13.4 h (norm < 5 h). This patient also showed a prolonged SBTT of 12.2 h (norm < 6 h). One patient with chronic constipation had a colonic transit time of 102.4 h (norm < 72 h). pH measurements showed a median gastric pH of 2.6 (IQR 1.6–3.2) (norm 0.5–5.1), a median pH of 7.1 (IQR 7.1–7.2) (norm 6.2–7.9) in the small bowel and a median pH of 6.6 (IQR 6.1–7.0) (norm 5.3–8.1) in the colon [17].

### Contractility parameters around gastric emptying

A detailed analysis of the contractility pattern was performed using the corresponding analysis software. This analysis has been used before in adults to define gastroparesis [8] as well as constipation by abnormal contractility in the respective region [11, 18]. Here, contractility parameters such as motility index (MI), contraction frequency (Ct/min) and area under curve (AUC) 1 h before (gastric contractility) and after gastric emptying (small bowel contractility) were calculated in all patients. As one patient showed a GET of 43 min (< 1 h), a shortened time window of 30 min was used to analyse contractility parameters in the gastric region (patient 5; Table 1). Contraction frequency (Ct/min) revealed a median of 0.6 (IQR 0.4–1.2) in the gastric region (norm > 0.48), 1.1 (IQR 0.5–1.8) in the small bowel (norm > 0.6) and 1.4 (IQR 1.2–1.8) in the colon (no normal values). The MI (MI = Ln (Ct *sum amplitudes + 1)) in the gastric and small bowel region revealed a median of 43.9 (IQR 31.8–70.4) (norm > 9.8) and 28.6 (IQR 18.0–41.9) (norm > 10.6), respectively. For the AUC (mmHg/s), we measured 2411 (IQR 1712–3638) (norm > 1358) in the gastric region and 1678 (IQR 1072–2220) (norm > 1456) in the small bowel region.

In total, the WMC test detected gastroparesis in two patients with prolonged GET (patients 3 and 9; Table 1). This was evaluated in at least one window (either gastric and/or small bowel window) of contractility parameters. For patient 3, this was evaluated by gastric contractility abnormalities, while small bowel contractility was normal. Patient 9 had normal gastric contractility parameters whereas small bowel contractility parameters were abnormal (Table 1, Fig. 1b). Interestingly, one patient (patient 6) with normal GET and normal SBTT showed gastric as well as small bowel contractility abnormalities (Table 1).

### Impact of WMC test results on diagnosis, further investigations and therapy

The WMC test showed delayed transit times in one or more regions in four patients. This new diagnostic information led to further diagnostic procedures and change in therapy (Table 2). All four patients reported symptoms that can be attributed to both upper and lower GI tract motility disorders. For example, a patient with gastroparesis had chronic abdominal pain and recurrent vomiting, and a patient with constipation had epigastric complaints. Three out of four patients showed prolonged transit times in more than one GI region.

Therapy of the four patients was modified by adding prokinetics and ursodeoxycholic acid or by intensifying laxative therapies. In half of the patients, further motility tests were not necessary. Overall, therapy led to improvement of symptoms in all four patients with delayed transit times (Table 2).

## Discussion

Although assessment of paediatric dysmotility in functional GI disease remains a challenge, the WMC test may be a valuable tool generating a motility profile of the entire GI tract. Abnormal regional transit times and divergent contractility patterns may help to explain dysmotility symptoms and diagnose motility disorders such as gastroparesis or slow transit colon.

There are only few data on the impact of new diagnostic information generated by the WMC test and further changes in therapy regimes in the paediatric population. Here, we additionally present clinical data on symptomatology with corresponding outcome after individual adjustment of therapy following performance of the WMC test (Table 2).

The impact of new diagnostic information generated by the WMC test and further changes in therapy regimes have not been described in a paediatric cohort so far. Four of nine patients showed abnormal transit times in different GI regions. Both patients with prolonged GET also had a delayed SBTT, and in one patient with constipation and prolonged CTT, SBTT was also delayed. Green et al. recently showed that all six patients with delayed SBTT had gastroparesis on the WMC test, whereas five patients with delayed SBTT had gastroparesis on scintigraphy [4]. Interestingly, this phenomenon has been found in adults as well: some patients with gastroparesis also showed delayed SBTT [19] and some patients with constipation had abnormalities in upper gut motility [20–22]. Kuo et al. [12] could show in a relevant proportion of adults that analysis of WMC data also revealed transit abnormalities in two or more GI regions. One can suggest that patients with dysmotility in one GI region often have other GI regions affected, which may contribute to the complexity of symptoms.

To assess upper GI motility and regional transit, ADM, gastric emptying scintigraphy and radiopaque marker studies are well-established diagnostic methods. However, ADM is an invasive test and gastric scintigraphy in not available in every paediatric gastroenterology centre and standardised protocols are often lacking for the paediatric population [4]. Radiopaque marker studies are also non-invasive tests but include exposure to radiation and only assess colonic transit. Performing only one of these examinations would not have been sufficient to detect all transit abnormalities in our patients with complex symptoms ultimately leading to the appropriate therapy.

In children, the WMC test revealed a sensitivity of 100% compared to scintigraphic gastric emptying studies in detecting gastroparesis [4]. Remarkably, the WMC test was able to detect even more contractile abnormalities than ADM either in the gastric or small bowel contractile window [4]. However, compared to ADM, the WMC has its limitations due to its single pressure sensor (Supplemental Fig. 1a) and free-floating nature. Abnormal contractility does not always lead to a delayed GET or SBTT but may contribute to the patient’s symptoms [4]. In line with this, we could show that one patient with chronic abdominal pain and irritable bowel syndrome had an abnormal contractility within the gastric as well as the small bowel region while having a normal GET and SBTT (Table 1, patient 6). This would argue for a high sensitivity of the WMC method to detect dysmotility, even when normal transit times are present, as described previously [4]. Although the clinical utility of pressure measurements by WMC in diagnosing motility disorders has to be elucidated, contractility data of WMC has been shown to be reliable in gastroparetic patients [9] as well as in subjects with constipation [23].

A limitation of our data series is its small sample size and its retrospective nature. To date, there is no approval for the diagnostic use in the paediatric population. Therefore, data in the paediatric population are very limited. The WMC test has been extensively studied in adults and has been approved by the US Food and Drug Administration to diagnose gastroparesis and constipation. Although the correlation between GI transit measured via WMC and scintigraphy studies has recently been shown in children [4], this still has to be confirmed in future research. Moreover, the diagnostic utility of detecting contractility abnormalities in patients with normal transit times is still unclear. These abnormalities alone do not prove an underlying pathology. Another limitation is the lack of reference values in paediatric patients. However, as we performed the WMC test only in adolescent patients, we consider the use of adult reference ranges to be appropriate.

In summary, we showed that the WMC test is a safe and minimally invasive procedure for paediatric patients providing relevant data on gut motility leading to individualised therapy. In four of our nine patients with functional GI symptoms, abnormal motility in either the upper GI tract leading to gastroparesis or the colon leading to constipation could be detected. This allowed individualised and optimised therapy for patients with functional GI disorders. The impact of new diagnostic information generated by the WMC test and further changes in therapy regimes have not been described in a paediatric cohort so far. The WMC test led to changes of therapy regimes in two out of nine patients with delayed GET as a prokinetic therapy with erythromycin or domperidone led to improvement or even resolution of symptoms (Table 2). Our results suggest that the WMC test is able to augment diagnostic opportunities for patients with functional GI disorders and suspected motility disturbances.

## Supplementary information

Below is the link to the electronic supplementary material.

Supplementary file1 (PDF 62 KB)

## Data Availability

All data and materials are included in the manuscript or uploaded as supplementary information.

## Abbreviations

ADM: Antroduodenal manometry
AUC: Area under the curve
CTT: Colonic transit time
Ct: Contractions per minute
GET: Gastric emptying time
GI: Gastrointestinal
IQR: Interquartile range
MI: Motility index
SBTT: Small bowel transit time
SLBTT: Small and large bowel transit time
WMC: Wireless motility capsule
WGTT: Whole gut transit time
