# Supplementary material for: Assessment of whole gut motility in adolescents using the wireless motility capsule test
Source: Eur J Pediatr. 2021 Nov 17;181(3):1197–204. doi: 10.1007/s00431-021-04295-6 (PMC8897340; doi:10.1007/s00431-021-04295-6)
Supplement: Supplementary file 1 — Supplementary file1 (PDF 62 KB) [file 431_2021_4295_MOESM1_ESM.pdf]

Supplemental Figure 1

a: Capsule dimensions and technical content
b: Flowchart of the standardised test procedure for performing a wireless motility capsule test

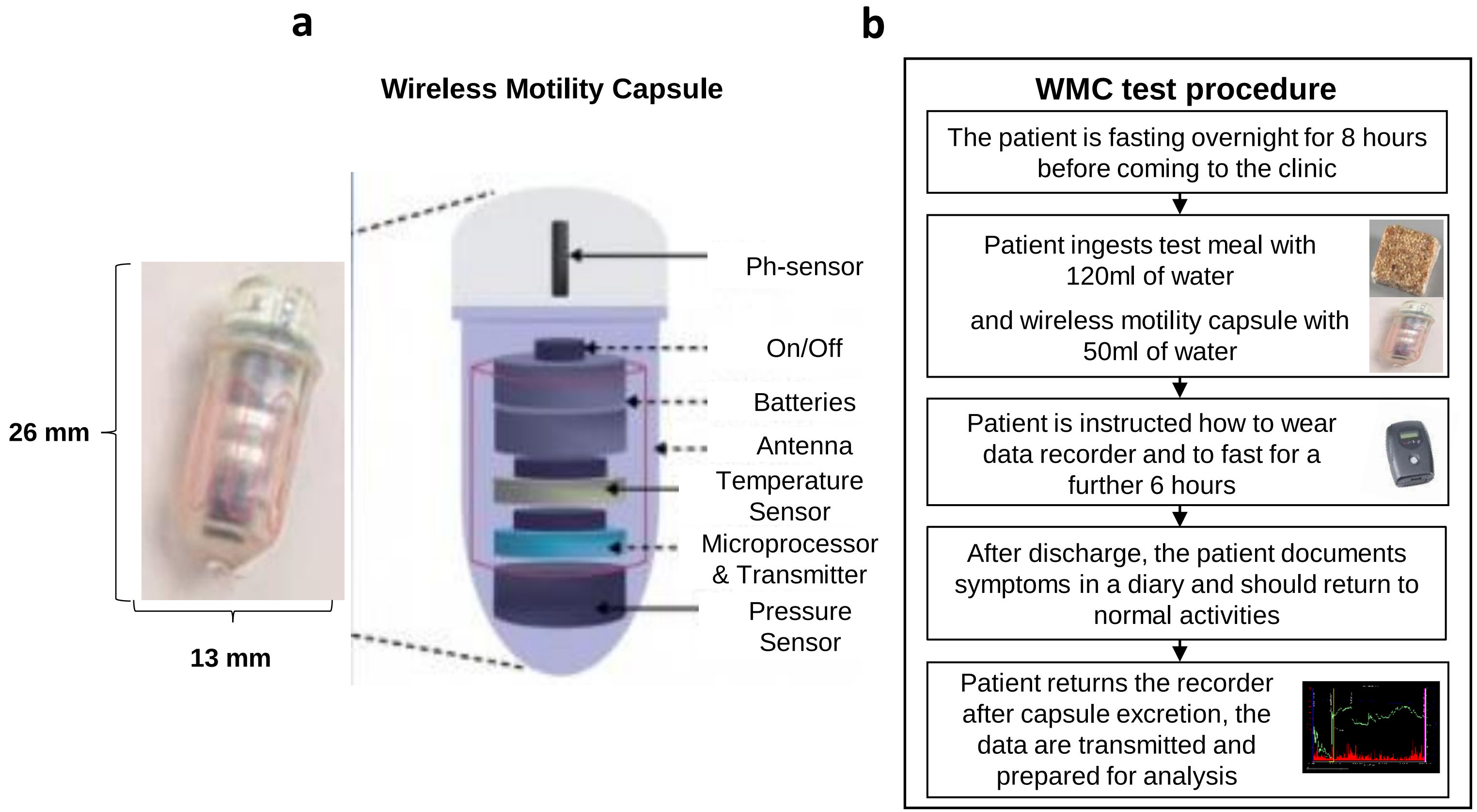

Assessment of Whole Gut Motility in Adolescents Using the Wireless Motility Capsule Test;
European Journal of Pediatrics,
Fritz T, Hünseler C, Broekaert I; Department of Paediatrics, Faculty of Medicine, University Children´s Hospital, University of Cologne, Cologne, Germany;
tanja.fritz@uk-koeln.de
